# Development of a Core Critical Care Data Dictionary With Common Data Elements to Characterize Critical Illness and Injuries Using a Modified Delphi Method

**DOI:** 10.1097/CCM.0000000000006595

**Published:** 2025-02-21

**Authors:** David J. Murphy, Wesley Anderson, Smith H. Heavner, Tamara Al-Hakim, Raul Cruz-Cano, Krzysztof Laudanski, Rishikesan Kamaleswaran, Omar Badawi, Heidi Engel, Jocelyn Grunwell, Vitaly Herasevich, Ashish K. Khanna, Keith Lamb, Robert MacLaren, Teresa Rincon, Lazaro Sanchez-Pinto, Andrea N. Sikora, Robert D. Stevens, Donna Tanner, William Teeter, An-Kwok Ian Wong, James L. Wynn, Xiaohan T. Zhang, Jerry J. Zimmerman, Vishakha Kumar, J. Perren Cobb, Karin E. Reuter-Rice

**Affiliations:** 1 Division of Pulmonary, Allergy, Critical Care and Sleep Medicine, Department of Medicine, Emory University, Atlanta, GA.; 2 Critical Path Institute, Tuscon, AZ.; 3 Society of Critical Care Medicine, Mount Prospect, IL.; 4 Department of Epidemiology & Biostatistics, Indiana University Bloomington, Bloomington, IN.; 5 Division of Critical Care Medicine, Department of Anesthesiology and Perioperative Medicine, Mayo Clinic, Rochester, MN.; 6 Department of Biomedical Informatics, Duke University, Durham, NC.; 7 National Evaluation System for Health Technology, Arlington, VA.; 8 Department of Rehabilitative Services, University of California San Francisco, San Francisco, CA.; 9 Department of Pediatrics, Emory University, Atlanta, GA.; 10 Department of Anesthesiology, Section on Critical Care Medicine, Wake Forest University, Winston-Salem, NC.; 11 Pulmonary Diagnostics & Respiratory Therapy Services, University of Virginia Medical Center, Charlottesville, VA.; 12 Department of Clinical Pharmacy, University of Colorado, Aurora, CO.; 13 School of Nursing, University of Massachusetts, Amherst, MA.; 14 Division of Health and Biomedical Informatics, Department of Preventive Medicine, Northwestern University, Evanston, IL.; 15 Department of Biomedical Informatics, University of Colorado School of Medicine, Aurora, CO.; 16 Department of Anesthsiology and Critical Care Medicine, Johns Hopkins University, Baltimore, MD.; 17 Department of Intensive Care and Resuscitation, Cleveland Clinic, Cleveland, OH.; 18 Department of Emergency Medicine, University of Maryland, Baltimore, MD.; 19 Division of Pulmonary, Allergy, and Critical Care Medicine, Department of Medicine, Duke University, Durham, NC.; 20 Department of Pediatrics, University of Florida, Gainesville, FL.; 21 Department of Medicine, Johns Hopkins University, Baltimore, MD.; 22 Division of Pediatric Critical Care Medicine, Department of Pediatrics, University of Washington, Seattle, WA.; 23 Division of Trauma, Emergency Surgery and Surgical Critical Care, Department of Surgery, University of Southern California, Los Angeles, CA.; 24 School of Nursing, Duke University, Durham, NC.

**Keywords:** adult, common data elements, intensive care, neonates, pediatric, standard definitions

## Abstract

**OBJECTIVES:**

To develop the first core Critical Care Data Dictionary (C2D2) with common data elements (CDEs) to characterize critical illness and injuries.

**DESIGN:**

Group consensus process using modified Delphi approach.

**SETTING:**

Electronic surveys and in-person meetings.

**SUBJECTS:**

A multidisciplinary workgroup of clinicians and researchers with expertise in the care of the critically ill and injured.

**INTERVENTIONS:**

The Delphi process was divided into domain and CDE portions with each composed of two item generation rounds and one item reduction/refinement rounds. Two in-person meetings augmented this process to facilitate review and consideration of the domains and by panel members. The final set of domains and CDEs was then reviewed by the group to meet the competing criteria of utility and feasibility, resulting in the core dataset.

**MEASUREMENTS AND MAIN RESULTS:**

The 23-member Delphi panel was provided 1833 candidate variables for potential dataset inclusion. The final dataset includes 226 patient-level CDCs in nine domains, which include anthropometrics and demographics (8), chronic comorbid illnesses (18), advanced directives (1), ICU diagnoses (61), diagnostic tests (42), interventions (27), medications (38), objective assessments (26), and hospital course and outcomes (5). Upon final review, 91% of the panel endorsed the CDCs as meeting criteria for a minimum viable data dictionary. Data elements cross the lifespan of neonate through adult patients.

**CONCLUSIONS:**

The resulting C2D2 provides a foundation to facilitate rapid collection, analyses, and dissemination of information necessary for research, quality improvement, and clinical practice to optimize critical care outcomes. Further work is needed to validate the effectiveness of the dataset in a variety of critical care settings.

**KEY POINTS**

**Question**: What domains and common data elements should be included in a core Critical Care Data Dictionary?

**Findings**: Using a modified Delphi process, a 23-member panel developed the first core Critical Care Data Dictionary to characterize critical illness and injury. The data dictionary includes nine domains and 226 common data elements, which can be applied to neonatal through adult patients.

**Meaning**: The dataset is intended to provide a foundation for the rapid collection, analysis, and dissemination of information necessary for research, quality improvement, and clinical practice to optimize critical care outcomes.

In the United States, critical care healthcare costs have been estimated to be 13% of hospital expenditures and have increased by 92% with a total cost of $108 billion U.S. dollars over a 10-year period (2000–2010) (1). Mortality rates exceed those of all other hospital care areas with one in five deaths occurring in the critical care setting (2). Strategies to improve patient outcomes and contain costs lie in identifying critical care research priorities, conducting innovative research, and translating evidence into practice. Accomplishing these goals depends upon robust data aggregation, analysis, and reporting (3).

The current state of critical care data infrastructure faces multiple limitations. Highly customizable electronic health record (EHR) platforms (e.g., Epic, Cerner, Meditech) promote fit-for-purpose interfaces for clinical practice, but also drive heterogeneity in data structure, limiting data usability for multi-institution clinical research (4–6). Harmonizing data from multiple sources is time-intensive and costly, and secondary sources (e.g., registries and repositories) often have limited interoperability and reusability (7, 8). These realities hampered the COVID-19 response by hindering the ability to rapidly establish the large-scale databases necessary to generate clinical knowledge (e.g., risk factors, symptom progression, pathophysiology, effective therapies, outcomes) within and across patient populations (9–11). Left unaddressed, these limitations will undoubtedly present challenges in future pandemics.

To address these limitations, the Society of Critical Care Medicine (SCCM) launched a workgroup within the Discovery Oversight Committee to identify gaps in critical care research and the strategies necessary to address those gaps. In 2022, Discovery, the Critical Care Research Network, developed a robust 5-year research agenda, which included the creation of the Discovery Data Science Campaign (DSC) (12). Within its large-scale data harmonization and data sharing initiative, the DSC recognized the need to develop a Critical Care Data Dictionary (C2D2) with standardized common data elements (CDEs) to facilitate the development of a robust data infrastructure, support, and perform pilot projects, implement the C2D2, and evaluate usability and efficacy. This article describes the results of a modified Delphi process used to develop the data dictionary with CDEs.

## METHODS

### Design

The Delphi method is a proven strategy for addressing crucial clinical inquiries lacking strong evidence (13–15). Specifically applied in healthcare, it harnesses expert opinions through iterative rounds of consensus and voting (16, 17). A “modified” Delphi enhances this process by integrating other strategies (e.g., in person meetings) to foster interactive discussions and accommodate new evidence, while maintaining the strengths of the traditional Delphi process (18).

**Figure 1.**
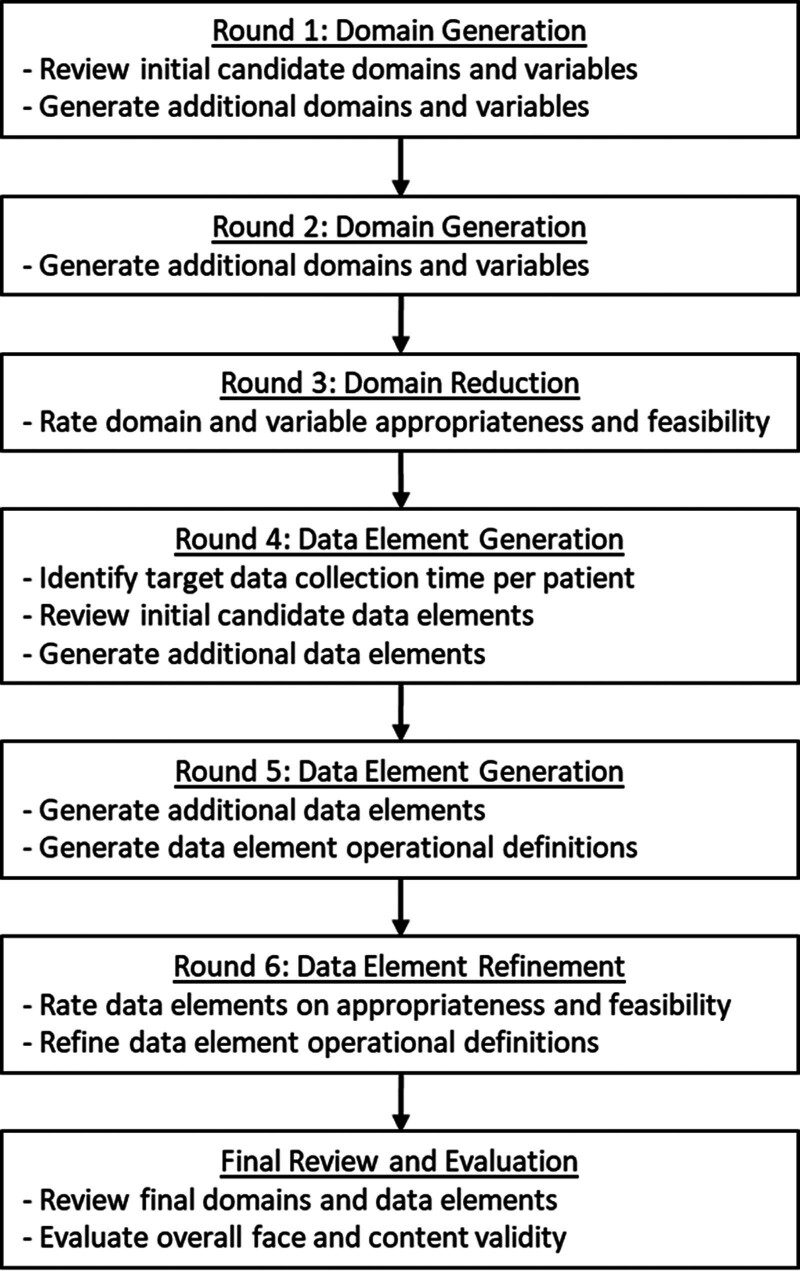
Flow diagram of consensus process.

We divided our consensus process into domain (i.e., high level category) and data element (i.e., specific measure), portions with each portion composed of two item generation rounds and one item reduction/refinement round (**Fig. 1**). Delphi rounds leveraged online surveys (Welphi.com, Lisbon, Portugal) to facilitate asynchronous participation, regardless of location. Members were requested to complete surveys within 2 weeks of receipt, receiving reminders 1 week after the initial distribution to encourage full participation across all rounds. The traditional Delphi process was augmented by two in-person meetings to facilitate review and consideration of the domains and data elements by panel members. The Delphi process was classified as nonhuman subjects research by the Emory University Institutional Review Board.

### Panel Participants

SCCM sought participants for a multidisciplinary panel comprising clinicians and investigators with extensive experience in acute and critical care across all age groups (**Supplementary Table 1**, http://links.lww.com/CCM/H679). Leadership also sought to include a broad range of nonclinical expertise, including basic science, clinical research, data science, informatics, and quality improvement. Experts were initially selected based on clinical or technical expertise to create a critical mass of greater than 20 for the modified Delphi process, with interest to include experts who had neonatal, pediatric, and adult clinical expertise, in addition to technical industry experts. There was also an emphasis to ensure gender balance and actively promote the inclusion of underrepresented minorities across all groups. All participants completed conflict of interest documentation, which was confirmed at each Delphi interval.

### Framework

Participants were guided by a framework (**Table 1**) throughout the process, outlining the concept, purpose, and operational characteristics of the common dataset. The dataset’s aim is to capture essential information about critical care patients upon their presentation to the ICU (e.g., age, diagnosis) and their clinical care details (e.g., medications, interventions, diagnostic tests), with outcomes assessed at facility discharge. Due to the varied resources available across critical care locations, the panel sought to design the initial dataset to enable manual data capture while also ensuring compatibility with automated extraction from an EHR. Furthermore, the panel sought to leverage prior work by incorporating existing composite disease severity scores and other data tools where feasible.

**TABLE 1.** Framework for Core Dataset and Data Attributes

Strategy
Support use across the human lifespan including neonatal, pediatric, and adult populations
Support use across the spectrum of clinical illness and injuries experienced by critically ill patients
Purpose
Capture information to determine adequacy and value of resource utilization
Provide information on patient outcomes across time
Characteristics
Feasible across all critical care settings
Extensible to provide project-specific information
Facilitate manual and electronic data abstraction
Real-time capture of information across spectrum of care
Data attribute: Four tiers of item categorization
Domain—high level categories of data (e.g., clinical assessment)
Subdomain—smaller group of variables within a domain (e.g., blood pressure)
Concept—specific measurement (e.g., MAP)
Common data element—concept with defined framework for collection including temporality and plausibility rules (e.g., lowest MAP in first 24 hr of ICU admission measured by cuff)

MAP = mean arterial pressure.

Additionally, the workgroup conceptualized four tiers of item categorization: 1) “domain,” high level categories of variables (e.g., vital signs); 2) “subdomain,” smaller group of variables within a domain (e.g., blood pressure); 3) “concept,” specific measurements or variables (e.g., mean arterial pressure [MAP]); and 4) “CDEs,” with defined framework for collection, including temporality and plausibility rules (e.g., lowest MAP in first 24 hr of ICU admission, measured by arterial line).

### Domain Focus (Rounds 1–3)

After agreeing to participate, panel members suggested data dictionaries that addressed relevant clinical and research activities. Biostatisticians combined these into an initial dataset, forming the basis for the Delphi study to identify critical care CDEs. Workgroup members with specific data science expertise then categorized these initial concepts into domains and subdomains, which provided the infrastructure for the initial dataset.

A virtual introductory meeting was held where the panel discussed the overarching framework, received an overview of the process, and reviewed the initial dataset for clarity regarding the next steps. During round 1, the panel received the initial dataset and were instructed to propose additional domains and associated subdomains. These additions were aggregated anonymously into a revised dataset, which was subsequently circulated among the group for round 2 of evaluation, where panelists were able to propose additional domains, subdomains, and concepts.

In round 3, panel members considered the significance and feasibility of each proposed domain and subdomain. Members then rated each domain and subdomain on a scale of 1–9 (1–3 being not important to the core dataset; 4–6 important but not critical; 7–9 critically important). Domains and subdomains rated as “critically important” by greater than 80% of the panel were advanced to be represented in the final dataset.

### Data Element Phase (Rounds 4–6)

The remaining rounds of the modified Delphi focused on generating and refining specific concepts. An in-person meeting was conducted to review the defined domains with associated subdomains and concepts extracted from the foundational data dictionaries in the context of the overarching framework. The panel was organized into multidisciplinary, domain-specific breakout groups to construct a list of candidate CDEs with associated operational definitions.

In round 4, the members were asked to identify the target data collection time per patient, review the initial candidate CDEs, and propose additional CDEs with associated operational definitions. The additional CDEs were aggregated and circulated to the panel for additional CDE proposals during round 5.

Round 6 began with an in-person meeting at SCCM Congress, where the panel reviewed and refined CDE operational definitions. Specifically, experts reviewed the data dictionary to determine whether existing CDEs were sufficient for each severity score or if additional concepts and CDEs were needed. The updated data dictionary was disseminated to panel members, who rated elements on importance and feasibility. Panelists were also given the option to further refine CDE operational definitions.

The final core dataset was reviewed by the panel for content clarity, construct validity, feasibility, and overall alignment with the project objective. Panelists voted electronically on final agreement of the resulting data dictionary with selected CDEs. An a priori consensus threshold of greater than 80% endorsement was set to complete the consensus process and finalize the dataset.

## RESULTS

### Participants

Of the 23 panel members, 91% work at academic medical centers, 9% work for government agencies, 4% are a part of nonprofit organizations, and 4% are employed in industry (**Table 2**). The panel included a broad range of educational backgrounds, including but not limited to master’s or PhD trained researchers (74%), physicians (57%), nurses (13%), and pharmacists (13%). The majority (87%) of the group had clinical expertise in critical care, with eight other clinical specialties (e.g., neonatal, pediatric, anesthesiology, emergency medicine). Panelists contributed a broad range of nonclinical expertise including clinical research (61%), data science (61%), quality improvement (48%), and clinical informatics (35%).

**TABLE 2.** Characteristics of Workgroup (*n* = 23)

Participant	*n* (%)
Employment type	
Academic medicine	21 (91)
Other nonprofit	2 (9)
Government	1 (4)
Industry	1 (4)
Education	
Research (MA, MS, PhD)	17 (74)
Physician (MD, DO)	13 (57)
Nursing (RN, NP)	3 (13)
Pharmacy (PharmD)	3 (13)
Respiratory therapy (RT)	2 (9)
Physical therapy (DPT)	1 (5)
Clinical specialty	
Critical care medicine	20 (87)
Pediatrics	4 (17)
Anesthesiology	2 (9)
Emergency medicine	2 (9)
Pulmonology	2 (9)
Internal medicine	1 (5)
Neonatology	1 (5)
Neurology	1 (5)
Surgery	1 (5)
Other expertise	
Clinical research	14 (61)
Data science	14 (61)
Quality improvement	11 (48)
Clinical informatics	8 (35)
Translational science	7 (30)
Implementation science	5 (22)
Clinical trialist	4 (17)
Bench science	3 (14)
Telehealth	2 (9)

DO = Doctor of Osteopathic Medicine, DPT = Doctor of Physical Therapy, MA = Master of Arts, MD = Medical Doctor, MS = Master of Science, NP = Nurse Practitioner, PharmD = Doctor of Pharmacy, PhD = Doctor of Philosophy, RN = Registered Nurse, RT = Respiratory Therapist.

a Percentages exceed 100%.

### Delphi Rounds

Initially, five existing data dictionaries were reviewed as prior work, with two being eliminated due to overlap or narrow focus (4, 19). Three data dictionaries from SCCM-sponsored studies—the Viral Infection and Respiratory Illness Universal Study COVID-19 Registry (VIRUS), Severe Acute Respiratory Infection Preparedness (SARI-PREP), and International Severe Acute Respiratory and Emerging Infection Consortium (ISARIC)—were retained and compiled into an initial pre-Delphi dataset, with 19 domains and 125 subdomains (**Table 3**) (20–22). After the first two rounds of domain generation, the dataset had increased to 20 domains, including 191 subdomains. After round 3 of the consensus process (item reduction), the panel identified nine domains and 36 subdomains as critically important to a minimal viable core dataset, and these were retained to inform the subsequent phases.

**TABLE 3.** Domain and Subdomain Results After the First Three Modified Delphi Rounds

Level	No. of Domains			Examples
	Initial (Round 0)	Item Generation (After Round 2)	Item Reduction (After Round 3)	
Domain	19	20	9	Diagnoses, outcomes
Subdomain	125	191	36	Weight, blood pressure

At the beginning of round 4, most of the group (57%) agreed that 10–30 minutes was the ideal time to manually collect basic patient-level data. The remaining (43%) of the group voted that more than 30 minutes was an ideal collection time. CDE generation and refinement in rounds 4–6 increased the total number of CDEs in the core data set from 104 to 226 (**Table 4**). Most CDEs were included in the diagnoses, diagnostic tests, and medication domains of the final dataset (61, 42, and 38, respectively). Several CDEs were added within a single concept due to different required assessment times (e.g., first 4 hr vs. first 24 hr) or different desired values (e.g., highest and lowest oxygen saturation within the first 24 hr of ICU admission). Upon final review, the a priori consensus threshold of greater than 80% was met with a final vote of 91% (*n* = 21) yes; 4.3% (*n* = 1) no; and 4.3% (*n* = 1) missing.

**TABLE 4.** Common Data Element Results After the Second Three Modified Delphi Rounds

Domain	No. of Common Data Elements		Examples
	Initial	Final	
Advanced directives	5	1	Last code status at 24 hr in ICU
Anthropometrics and demographics	10	8	Age, sex, first documented weight
Chronic comorbid illnesses	20	18	Mild liver disease, dementia
Diagnoses	3	61	Asthma, necrotizing enterocolitis, trauma
Diagnostic tests	25	42	Platelet count (low), WBC count (high)
Interventions	3	27	Any invasive mechanical ventilation
Medications	21	38	Vancomycin, epinephrine infusion (maximum)
Objective assessments	10	26	Mean arterial blood pressure (low)
Outcomes and hospital course	7	5	Hospital discharge disposition
Total	**104**	**226**	

### Final Core Dataset

Overall, the final core dataset included nine domains and 226 CDEs, reflecting the spectrum of clinical illness and injuries experienced by critically ill patients across the human lifespan. The dataset includes a range of individual CDEs important to general critical care populations (e.g., invasive mechanical ventilation support duration, gastrointestinal bleeding) and those more focused on specific ICU patient populations (e.g., necrotizing enterocolitis). Furthermore, the inclusion of select social determinants of health elements (e.g., area deprivation index) enables the evaluation of social factors on a broad range of critical illness and injury (23–25).

Beyond the individual elements, the C2D2 supports multiple disease severity scores: Acute Physiology and Chronic Health Evaluation (APACHE) II (26, 27), APACHE III (28), Charlson Comorbidity Index (CCI) (29), Medication Regimen Complexity-ICU (30, 31) scoring tool, pediatric Sequential Organ Failure Assessment (SOFA) (32–34), neonatal SOFA (35), Pediatric Risk of Mortality III (36, 37), Pediatric Index of Mortality 3 (38–40), and SOFA (41–44). The majority of CDEs (181 [80%]) contributed to at least one disease severity score (**Supplementary Table 2**, http://links.lww.com/CCM/H679). The full data dictionary is available in an **online spreadsheet** (http://links.lww.com/CCM/H703).

## DISCUSSION

We report the development of a core clinical dataset that provides a standard foundation and template for use in critical care research and quality improvement: the C2D2. The use of a standardized consensus approach, based on the Delphi process, allowed the assembled multidisciplinary workgroup to reach a consensus on the most important information to collect to provide a better understanding of the patient-level data, although still meeting the goal of feasibility for the critical care space. The C2D2 includes elements that characterize disease phenotypes and severity of illness. The dataset is designed to be applicable across the entire patient-care spectrum, including the neonatal, pediatric, and adult populations, in both the academic and community hospital settings. The dataset also provides insights into critical care diagnostics and treatments employed. Finally, the dataset fosters the evaluation of resource matching, critical infrastructure, and operational performance.

Our project highlights the potential synergies achievable by integrating the C2D2 core dataset with data modernization techniques. The incorporation of advanced data collection, management, and analysis methods is poised to amplify the scope and depth of information captured in critical care environments. By coupling these strategies with the stringent definitions established for CDEs, our approach enhances the opportunity to leverage large multisite data capture and data translation, a foundational goal of SCCM’s Discovery Research Network. We also expect that C2D2 can be coupled with other data collection tools for quality improvement, such as the new SCCM Center of Excellence Program reporting standard, to benchmark ICU Liberation Bundle performance. Additionally, C2D2 facilitates the exploration of therapeutics and the formulation of hypotheses under research emergency conditions (such as pandemics) and across diverse domains, including neonatology and pediatrics (S. F. Heavner, unpublished observations, 2025).

The development of C2D2 marks a pivotal starting point in ensuring consistency, interoperability, and clarity in data management across various critical care systems and studies (S. F. Heavner, unpublished observations, 2025). It addresses crucial aspects by clearly defining each data element using standardized terminology, thereby eliminating ambiguity in interpretation. C2D2 assigns unique identifiers to facilitate efficient tracking and referencing across different systems, accompanied by clear descriptions of each CDE’s purpose and relevance. Furthermore, it specifies the data type and format for consistent representation and handling, supporting enforced consistency in data entry, analysis, integrity, and coherence (45, 46).

A strategic imperative is to ensure easy access to C2D2 for all stakeholders while offering comprehensive documentation to aid users in understanding and correctly applying the data elements. One proposed method is using a cloud-based data collection platform as part of SCCM’s datahub initiative. Additionally, this initiative lays the groundwork for aligning C2D2 with relevant metadata standards, such as those established by the Clinical Data Interchange Standards Consortium (47), Health Level Seven International, and Fast Healthcare Interoperability Resources, to improve interoperability and streamline data sharing across systems and studies (48, 49). By tackling these factors, C2D2 can initiate the advancement of consistency, interoperability, and efficiency in data management and analysis processes.

Limitations to this work warrant consideration and future investigation. First, it is essential to acknowledge that the use of the C2D2 core dataset in our study has not been formally validated. While our findings demonstrate its potential benefits, further feasibility assessments across diverse settings are imperative. For instance, the charge from the SCCM DSC specifically includes testing C2D2 in a SCCM Discovery-sponsored study. This DSC pilot study will be multicentered and yield not only important scientific findings related to its study questions but also C2D2 usability and feasibility information to help drive revisions to the data dictionary for use both in the United States and internationally.

Second, despite the advancements made in this study, it is essential to acknowledge that our work is not exhaustive. We recognize that further enhancements and expansions are both warranted and welcomed. To address this, we have initiated the establishment of additional data cassettes that will augment the core dataset in future iterations, including expanded neonatal and pediatric CDEs. Additionally, the standardized taxonomy of patient-level data in the C2D2 can facilitate linking to broader structural/organizational characteristics (e.g., workforce, facilities, costs) captured in U.S. and international databases (e.g., American Hospital Association’s Annual Survey, Centers for Medicare & Medicaid Services [CMS] Hospital Provider Cost Report, World Health Organization). These forthcoming additions will ensure the continued international relevance and utility of the dataset in evolving critical care landscapes.

In conclusion, our study provides a testable C2D2 core dataset, the use of which is aimed to improve patient outcomes, develop innovative treatments, implement new research findings, inform quality improvements, and identify critical care research priorities. Planned initiatives will next focus on validating C2D2, along with integrating data modernization techniques, and expanding the dataset to accommodate emerging needs. By addressing these considerations, we can foster a robust and comprehensive framework for data-driven decision-making and innovation in critical care.

## ACKNOWLEDGMENTS

We thank the contributions of the Discovery Oversight Committee in their review of this article.

## Supplementary Material




