# Supplementary material for: Development of a Core Critical Care Data Dictionary With Common Data Elements to Characterize Critical Illness and Injuries Using a Modified Delphi Method
Source: Crit Care Med. 2025 Feb 21;53(5):e1045–54. doi: 10.1097/CCM.0000000000006595 (PMC12047641; doi:10.1097/CCM.0000000000006595)
Supplement: Supplementary file 1 [file ccm-53-e1045-s001.docx]

**Title:** *Development of a Core Critical Care Data Dictionary (C2D2) with Common Data Elements to Characterize Critical Illness and Injuries* using a modified Delphi Method

**Authors:**

David J. Murphy, MD PhD FCCM^1^; Wesley Anderson, RN PhD^2^; Smith Heaver, PhD^2^; Tamara Al-Hakim, MD^3^; Raul Cruz-Cano, PhD^4^; Krzysztof Laudanski, MD PhD^5^; Rishikesan Kamaleswaran, PhD^6^; Omar Badawi, PharmD FCCM^7^; Heidi Engel, DPT^8^; Jocelyn Grunwell, MD^1^; Vitaly Herasevich, MD PhD FCCM^5^; Ashish K. Khanna, MD FCCM^9^; Keith Lamb, RRT FCCM^10^; Robert MacLaren, PharmD MCCM^11^; Teresa Rincon, RN PhD FCCM^12^; Lazaro Sanchez-Pinto, MD^13^; Andrea N. Sikora PharmD^10^; Robert D. Stevens, MD FCCM^14^; Donna Tanner, RRT^15^; William Teeter, MD^16^; An-Kwok Ian Wong, MD^6^; James L. Wynn, MD^17^; Xiaohan T. Zhang, MBBS^14^; Jerry J. Zimmerman, MD FCCM^18^; Vishakha Kumar,MD^3^; J. Perren Cobb, MD^19^; & Karin E. Reuter-Rice, PhD NP FCCM^6^

**Institutions:** 1. Emory University, Atlanta, GA; 2. Critical Path Institute, Tuscon, AZ; 3. Society of Critical Care Medicine, Mount Prospect, IL; 4. Indiana University Bloomington, Bloomington IN; 5. Mayo Clinic, Rochester, MN; 6. Duke University, Durham, NC; 7. National Evaluation System for Health Technology, Arlington, VA; 8. University of California San Francisco, San Francisco, CA; 9. Wake Forest University, Winston-Salem, NC; 10. University of Virginia, Charlottesville, VA; 10. University of Colorado, Aurora, CO; 11. University of Massachusetts, Amherst, MA; 12. Northwestern University, Evanston, IL; 14. Johns Hopkins University, Baltimore, MD; 15. Cleveland Clinic, Cleveland, OH; 16. University of Maryland, Baltimore, MD; 17. University of Florida, Gainesville, FL; 18. University of Washington, Seattle, WA; 19. University of Southern California, Los Angeles, CA

**Table of Contents:**

[Supplementary Table 1. Expert Panel Members 3](#_Toc184242015)

[Supplementary Table 2. Data elements by disease severity score 4](#_Toc184242016)

## Supplementary Table 1. Expert Panel Members

| **Full Name** | **Organization** |
| --- | --- |
| Omar Badawi, MPH, PharmD, FCCM | Telemedicine and Advanced Technology Research Center |
| J. Perren Cobb, MD, FACS, FCCM | Keck Medical Center of USC |
| Heidi J. Engel, PT, DPT | University of California San Francisco Medical Center |
| Jocelyn R. Grunwell, MD, PhD | Emory University/Children's Healthcare of Atlanta Egleston |
| Smith F. Heavner, PhD, RN | Critical Path Institute |
| Vitaly Herasevich, MD, PhD, FCCM | Mayo Clinic |
| David J. Murphy, MD, PhD, FCCM | Emory University School of Medicine |
| Rishikesan Kamaleswaran, PhD | Emory University School of Medicine |
| Keith D. Lamb, RRT, RRT-ACCS | University of Virginia |
| Krzysztof Laudanski, MD, PhD, FCCM | Mayo Clinic |
| Robert MacLaren, MPH, PharmD, MCCM | University of Colorado Skaggs School of Pharmacy |
| Karin Reuter-Rice, PhD, NP, FAAN, FCCM | Duke University |
| Teresa A. Rincon, PhD, RN, FCCM | University of Massachusetts School of Medicine |
| L. Nelson Sanchez-Pinto, MD, MBI | Ann & Robert H. Lurie Children's Hospital of Chicago |
| Andrea Sikora, BCCCP, PharmD, MSCR | University of Georgia College of Pharmacy |
| Robert David Stevens, MD, FCCM | Johns Hopkins University School of Medicine |
| Donna Tanner, RRT, MBA, RRT-ACCS | Cleveland Clinic |
| William Teeter, MD | University of Maryland |
| An-Kwok Ian Wong, MD, PhD | Duke University Medical Center |
| James L. Wynn, MD | University of Florida |
| Tanner Zhang, MBBS, MS | Johns Hopkins University School of Medicine |

## Supplementary Table 2. Data elements by disease severity score

| **Domains** | **APACHE II** | **CCI** | **MRC- ICU** | **PIM3** | **PRISM III** | **PSOFA** | **SOFA** | **None** |
| --- | --- | --- | --- | --- | --- | --- | --- | --- |
| Anthropometrics & demographics |  | 1 |  | 1 | 1 | 1 |  | 7 |
| Chronic comorbid illnesses | 6 | 18 |  |  |  |  |  |  |
| ICU stay… |  |  |  |  |  |  |  |  |
| Advanced directives |  |  |  |  |  |  |  | 1 |
| Diagnoses^a^ | 46 |  |  | 13 |  |  |  | 12 |
| Diagnostics | 14 |  |  | 4 | 14 | 4 | 4 | 12 |
| Interventions | 19 |  | 4 | 2 |  | 1 | 1 | 3 |
| Medications |  |  | 32 |  |  | 4 | 4 | 2 |
| Objective assessments | 10 |  |  | 6 | 6 | 7 | 8 | 4 |
| Hospital course & outcomes |  |  |  | 1 |  |  |  | 4 |
| **Total** | **95** | **19** | **36** | **27** | **21** | **17** | **17** | **45** |

^a^ Diagnoses are captured at increased granularity compared with specific severity scores. Counts of data elements reflect the number of concepts and/or common data elements which may be related to, or helpful in deriving, each severity score.
